# Benzodiazepines prescribing in insomnia : Between practice and guidelines

**DOI:** 10.1192/j.eurpsy.2021.1279

**Published:** 2021-08-13

**Authors:** M. Lagha, U. Ouali, F. Nacef

**Affiliations:** Department Of Psychiatry A, Razi hospital, Manouba, Tunisia

**Keywords:** Benzodiazepines, psychiatry, Insomnia, Prescribing

## Abstract

**Introduction:**

Benzodiazepines (BZD) are psychotropic drugs prescribed in psychiatry for their anxiolytic, hypnotic and sedative properties. Several guidelines aimed to limit the chronic use of BZDs. However, BZDs prescribing that does not comply with international recommendations remains widespread, estimated in France at 20% for hypnotic BZDs.

**Objectives:**

The aims of our study were to evaluate BZDs prescribing practices in the treatment of insomnia and to assess their compliance with international recommendations.

**Methods:**

This is a cross-sectional study conducted through a Google-forms self-administered questionnaire,intended for psychiatrists and psychiatric residents, over a period of two months, from April 1 to May 31, 2019.

**Results:**

One hundred physicians practicing in psychiatry answered our questionnaire. The response rate was 28%. Four BZDs are recommended for the treatment of insomnia, none of which is available in Tunisia. Almost the third of the participants did not systematically look for signs of sleep apnea syndrome before treating an insomnia (30.5%). For treating insomnia, the majority of the participants began by indicating hygieno-dietetic rules (64%), 4% prescribed directly a BZD. Cognitive behavioral therapy was not indicated at all by the particiants. The maximum duration of prescribing BZDs in insomnia was 4 weeks in 20% of cases, and more than 4 weeks in 38% of cases. Among the participants, 41% prescribed BZDs for the treatment of chronic insomnia.

**Conclusions:**

Insomnia appear to be badly managed and early drug prescribing is frequent. These practices do not comply with the recommendations of good practice and increase the risk of dependance and other side effects.

